# Effects of Glycyrrhizin (GL) Supplementation on Survival, Growth Performance, Expression of Feeding-Related Genes, Activities of Digestive Enzymes, Antioxidant Capacity, and Expression of Inflammatory Factors in Large Yellow Croaker (*Larimichthys crocea*) Larvae

**DOI:** 10.1155/2022/5508120

**Published:** 2022-12-01

**Authors:** Yuntao Wang, Wenxuan Xu, Jianmin Zhang, Jiahui Liu, Zhen Wang, Yongtao Liu, Kangsen Mai, Qinghui Ai

**Affiliations:** ^1^Key Laboratory of Aquaculture Nutrition and Feed (Ministry of Agriculture and Rural Affairs), Key Laboratory of Mariculture (Ministry of Education), Ocean University of China, 5 Yushan Road, Qingdao, Shandong 266003, China; ^2^Laboratory for Marine Fisheries Science and Food Production Processes, Qingdao National Laboratory for Marine Science and Technology, 1 Wenhai Road, Qingdao, Shandong 266237, China

## Abstract

A 30-day feeding trial was conducted to determine the effects of dietary glycyrrhizin (GL) on survival, growth performance, expression of feeding-related genes, activities of digestive enzymes, antioxidant capacity, and expression of inflammatory factors of large yellow croaker larvae with an initial weight of 3.78 ± 0.27 mg. Four 53.80% crude protein and 16.40% crude lipid diets were formulated with supplementation of 0%, 0.005%, 0.01%, and 0.02% GL, respectively. Results indicated that larvae fed diets with GL had higher survival rate and specific growth rate than the control (*P* < 0.05). Compared with the control, the mRNA expression of orexigenic factor genes including neuropeptide Y (*npy*) and agouti-related protein (*agrp*) were significantly increased in larvae fed the diet with 0.005% GL, while the mRNA expression of anorexigenic factor genes including thyrotropin-releasing hormone (*trh*), cocaine and amphetamine regulated transcript (*cart*), and leptin receptor (*lepr*) were significantly decreased in larvae fed the diet with 0.005% GL (*P* < 0.05). The trypsin activity in larvae fed the diet with 0.005% GL was significantly higher than the control (*P* < 0.05). The alkaline phosphatase (AKP) activity in larvae fed the diet with 0.01% GL was significantly higher than the control (*P* < 0.05). A clear increase of total glutathione (T-GSH) content, activities of superoxide dismutase (SOD), and glutathione peroxidase (GSH-Px) was observed in larvae fed the diet with 0.01% GL compared with the control (*P* < 0.05). Moreover, the mRNA expression of interleukin-1*β* (*il-1β*) and interleukin-6 (*il-6*) (proinflammatory genes) in larvae fed the diet with 0.02% GL were significantly lower than the control (*P* < 0.05). In conclusion, the supplementation of 0.005% -0.01% GL could enhance the expression of orexigenic factor genes, activities of digestive enzymes and antioxidant capacity, ultimately improving the survival, and growth performance of large yellow croaker larvae.

## 1. Introduction

Glycyrrhizin (GL), also known as glycyrrhizic acid (GA), is an important effective component of licorice, which is a traditional Chinese herbal medicine [1–3]. GL is also recognized as a natural green sweetener, and the sweetness of GL can reach hundreds of times that of sucrose [4–6]. In recent years, the pharmacological action of GL such as liver protection [7–9], anticancer [10, 11], and anti-inflammatory [12–15] have been widely recognized.

Several studies indicated that many main components of Chinese herbal medicines have the advantages of natural, nontoxic, and pollution-free and could be added into animal diets to increase the survival and growth performance of cultured animals. [16–18]. In recent years, studies have proved that GL could promote the growth performance, anti-inflammatory capacity, and innate immunity in the study of poultry and mammalian species [19, 20]. However, the application of GL in aquatic diets has been rarely reported. Results of the study in white shrimp (*Litopenaeus vannamei*), Asian seabass (*Lateolabrax maculatus*), and blunt snout bream (*Megalobrama amblycephala*) prove that GL could improve the anti-inflammatory capacity [21–23]. In particular, Harikrishnan et al. found that different dosages of GL could improve the growth, activity of digestive-antioxidant, and innate-adaptive defense of silver carp (*Hypophthalmichthys molitrix*) [24].

Because of the high nutritional and economic value, large yellow croaker (*Larimichthys crocea*) is popular in China [25, 26]. Large yellow croaker larvae are in the nutritional transition period from endogenous nutrition to exogenous nutrition [27, 28]. The larvae already have vision, smell, and taste when they ingested for the first time [29, 30]. However, the weak food intake capacity, immature digestive system, and poor stress resistance of larvae could lead to high mortality. [31–33]. At present, finding suitable feed additives to improve the appetite, digestion, absorption, and immunity of fish larvae is an important nutrition strategy to improve the survival rate of larvae. [34, 35].Though GL has good application prospects and development potential, there are no reports of GL found in the case of fish larvae farming. Therefore, the purpose of this study was to reveal whether GL supplementation could increase the survival, growth performance, expression of feeding-related genes, activities of digestive enzymes, antioxidant capacity, and expression of inflammatory factors in large yellow croaker larvae.

## 2. Materials and Methods

### 2.1. Feed Ingredients and Diet Formulation

In this trial four isonitrogenous (53.80% crude protein) and isolipidic (16.40% crude lipid) microdiets were formulated with the supplementing levels of 0%, 0.005%, 0.01%, and 0.02% of GL, respectively (Table 1). Pure GL (above 99.0%) were purchased from Qingdao kaitaike industry and Trade Co., Ltd in Qingdao, China.

Ingredients were ground into fine powder through 150 *μ*m nylon mesh. Then, the mesh of all ingredients was thoroughly mixed with water and fish oil to produce stiff dough. Extrude and round the dough with axial single screw spherical extruder and spheronization to obtain the pellet. Then the pellet was dried at 60°C in a constant temperature for 24 hours. After drying, the microdiets were ground into 250 to 380 *μ*m and 380 to 500 *μ*m particle sizes. Subsequently, the microdiets were packed in transparent and sealed plastic bags, respectively, and stored at -20°C until use. The particle size of the formulated diets ranged from 250 to 380 *μ*m for fish from 15 to 25 days after hatch (DAH) and 380 to 500 *μ*m for fish thereafter.

### 2.2. Feeding Trial

All larvae in this trial were purchased from Marine and Fishery Science and Technology Innovation Base, Zhejiang, China. Before the feeding trial, the larvae were acclimatized for 3 days by feeding live copepods and microdiets to adapt to the trial feed. Larvae were fed with rotifers (*Brachionus plicatilis*) (3-7 DAH), brine shrimp (*Artemia nauplii*) (5-10 DAH), copepods (*Calanus sinicus*) (8-14 DAH), and microdiets from (12-14 DAH). During the experimental period of 15 to 45 DAH, larvae were completely fed with experimental diet, respectively. Larvae with the initial weight of 3.78 ± 0.27 mg and the initial body length of 5.98 ± 0.39 mm were randomly distributed into 12 breeding plastic tanks (water volume 220 L), and each tank had 2500 larvae. Each diet was randomly assigned to triplicate cages (3 biological repetitions). During the experiment, larvae were fed seven times (06 : 30, 09 : 30, 12 : 30, 15 : 30, 18 : 30, 21 : 30, and 23 : 30) daily. The water temperature was maintained between 23 to 25°C by heating rods and temperature controllers, the pH was maintained between 7.80 to 8.20 and the salinity was maintained between 23 to 27 g/L by renewed water in tanks. The concentration of dissolved oxygen in aquaculture waters should be kept at about 6 mg/L by the gas stones. The daily aquaculture water renewed was maintained between 100 to 200% in each tank.

### 2.3. Sample Collection

Before the feeding trial, fifty larvae of 15 DAH were collected to measure the initial body length (IBL), initial body weight (IBM) by vernier calipers, and microbalance. After the feeding trial, the survival rate (SR) was calculated by counting the surviving larvae in each tank. Meanwhile fifty larvae of 45 DAH were collected to measure the final body length (FBL) and final body weight (FBL) by vernier calipers and microbalance. The remaining larvae were sampled after 24 hours of fasting to the empty intestine. Thirty larvae from each tank were dissected on ice to separate the head segment (HS) and visceral mass (VM) containing a crude mixture of pancreas, liver, heart, spleen, and intestine. The HS and VM were put into 2.0 ml RNase-free Cryogenic Vials then immediately frozen in liquid nitrogen for gene expression assays. The pancreatic segments (PS) and intestine segment (IS) were separated maintained at 0°C under a dissecting microscope and placed on a glass plate for the assay of activities of digestive enzymes. The remaining larvae in each tank were immediately frozen for body composition analysis.

### 2.4. Analytical Methods

#### 2.4.1. Body and Diet Composition Analysis

The samples of diet and larvae were laid inside an oven at 105°C for about 24 hours and dried to constant weight for moisture calculation. The crude protein and lipid contents of diet samples and larvae samples were measured by the method of AOAC (2003) with automatic Kjeldahl apparatus (Kjeltec TM 8400, FOSS, Tecator, Sweden) and Soxhlet extraction apparatus (B-801, Switzerland).

#### 2.4.2. Activities of Digestive Enzymes Assay

About 0.1 g PS or IS of larvae were weighed, separately homogenized in 1 ml PBS and centrifugated with a high-speed freezing centrifuge. The supernatant was prepared for the assay of *α*-amylase, trypsin, and lipase. The brush border membranes (BBM) of the intestine of larvae were extracted by the method of Crane et al. [36]. The BBM were prepared for the determination of alkaline phosphatase activity (AKP) and leucine-aminopeptidase (LAP). Activities of *α*-amylase, trypsin, and LAP were detected by a colorimetry, while activities of lipase and AKP were detected by a microplate reader. The unit of *α*-amylase was (U/mg·prot), 1 U/mg · prot was defined as 10 mg starch hydrolyzed per mg protein at 37°C for 10 minutes. The unit of trypsin was (U/mg · prot), 1 U/mg · prot was defined as producing 1 *μ*g of tyrosine at 37°C for 1 minute. The unit of lipase was (U/g · prot). 1 U/g·prot was defined as in this reaction system. 1 *μ*mol substrate was consumed by reacting with the substrate for 1 minute. The unit of AKP was (K/g · prot). 1 K/g·prot was defined as 1 mg phenol produced per g protein at 37°C for 15 minutes. The unit of LAP was (U/L. 1 U/L) was defined as 1 nmol p-nitroaniline produced per L at 37°C for 1 minute. Activities of *α*-amylase, trypsin, lipase, and AKP were determined in strict accordance with the instructions of assay kit (Nanjing Jiancheng Institute of Biological Engineering, China), and the activity of LAP was determined by the method of Maroux et al. [37]. Three replicates were made for each sample.

#### 2.4.3. Activity of Antioxidant and Innate Immune Enzymes Assay

About 0.1 g VM of larvae were weighed, separately homogenized in 1 ml PBS and centrifugated with a high-speed freezing centrifuge. The supernatant was prepared to detect activities of antioxidant and innate immune enzymes. Activities of superoxide dismutase (SOD), total antioxidant capacity (T-AOC), content of malondialdehyde (MDA), total glutathione (T-GSH), glutathione (GSH), and nitric oxide (NO) were detected by a microplate reader, activities of catalase (CAT), glutathione peroxidase (GSH-Px), total nitric oxide synthase (T-NOS), and inducible nitric oxide synthase (iNOS) was detected by a colorimetry. The unit of SOD was (U/mg · prot). The amount of enzyme corresponding to the sod inhibition rate of 50% in this reaction system was 1 activity unit. The unit of CAT was (mmol/g · prot). 1 mmol/g · prot was defined as the amount of 1 *μ*mol H_2_O_2_ decomposed per mg protein per second. The unit of T-AOC was (mmol/g · prot), which was defined as total antioxidant per g protein. The unit of MDA was (nmol/mg · prot), which was defined as the content of MDA per mg protein. The unit of GSH-Px was (U/mg · prot). 1 U/mg · prot was defined as reducing 1 *μ*mol/L GSH in the reaction system (deduct nonenzymatic reaction) per mg protein for 1 minute. The unit of T-GSH and GSH was (*μ*mol/L). The unit of NO was (*μ*mol/g · prot), which was defined as the content of NO per g protein. The unit of T-NOS and iNOS was (U/mg · prot). 1 U/mg · prot was defined as producing 1 nmol NO per mg protein for 1 minute. All of above enzymes were determined in strict accordance with the instructions of assay kit (Nanjing Jiancheng Institute of Biological Engineering, China). Three replicates were made for each sample.

### 2.5. RNA Extraction and RT-qPCR

About 0.03 g HS or VM of larvae were weighed and separately added into 1 ml Trizol reagent (Takara, Japan). Total RNA was extracted according to the manufacturer's protocol. The integrity of RNA was evaluated by electrophoresis. After that, the total concentration of extracted RNA was measured by Nano Drop® 2000 spectrophotometer (Thermo Fisher Scientific, USA). Then, the RNA was reversed to cDNA with Prime Script-RT reagent kit (Takara, Japan). Finally, the RT-qPCR was carried out by a quantitative thermal cycler (CFX96TM Real-Time System, BIO-RAD, USA). The reference gene in this experiment was *β-actin*. All of the primer sequences in this study were synthesized or designed on the basis of the published sequences from Huang et al. [38], Wu et al. [39], and GenBank (Table 2). The reaction system of qPCR was 20 *μ*l in total containing 10 *μ*l SYBR® Premix Ex Taq™ II (Takara, Japan), 7 *μ*l of RNase-free water, 2 *μ*l of cDNA, 0.5 *μ*l of upstream, and downstream primers. The program of qPCR was as follows: 95°C for 2 mins, followed by 39 cycles of 95°C for 10 s, 59°C for 10 s, and 72°C for 20 s. The mRNA expression of genes was calculated with the previous method (2^–*ΔΔ*CT^) [40]. Three replicates were made for each sample.

### 2.6. Calculation and Statistical Analysis

(1) $$\begin{array}{c} \text{Specific growth rate }\left(\text{SGR},\%\text{day}-1\right)=\text{Ln }\left(\text{final fish weight}\right)-\text{Ln }\left(\text{initial fish weight}\right)\times \frac{100}{\left(\text{the experimental duration}\right)}.\\ \text{Survival rate}\left(\text{SR},\%\right)=100\times \frac{\left(\text{final larvae number}\right)}{\text{initial larvae number}},\\ \text{Weight gain rate}\left(\text{WGR},\%\right)=\frac{\left(\text{final fish weight}-\text{initial fish weight}\right)}{\text{initial}}\text{fish weight}\times 100. \end{array}$$

SPSS Statistics 19.0 was used to conduct all data in this study, and then one-way analysis of variance (ANOVA) and Tukey's test were used to analyze the data. The significance level was selected as *P* <0.05. All results were presented as means ± S.E.M (standard error of the mean).

## 3. Results

### 3.1. Survival, Growth Performance and Body Composition

With increasing GL supplementation, the SR, FBL, FBW, WGR, and SGR in larvae increased firstly and then decreased (Table 3). The SR of larvae fed diets with GL was significantly higher than larvae fed the control diet (*P* < 0.05). Compared with the control, the FBL, FBW, WGR, and SGR were significantly increased in larvae fed diets with GL (*P* < 0.05). Furthermore, the FBW and SGR of larvae fed the diet with 0.005% GL were significantly higher than larvae fed diets with 0.01% and 0.02% GL (*P* <0.05). There were no significant differences among dietary treatments in the body composition (*P* > 0.05) (Table 4).

### 3.2. The mRNA Expression of Orexigenic and Anorexigenic Factor Genes

**Figure 1:**
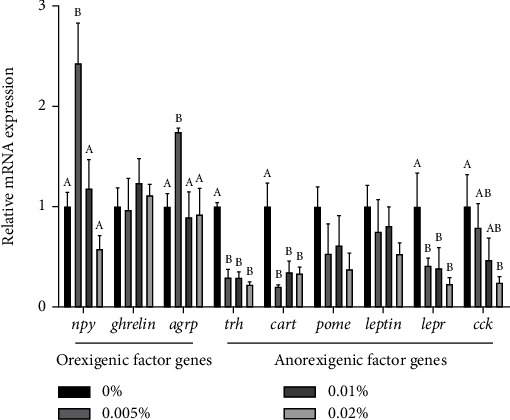
Expression of genes related to orexigenic and anorexigenic factor of large yellow croaker larvae. The *npy* (neuropeptide Y), *agrp* (agouti-related protein), *trh* (thyrotropin-releasing hormone), *cart* (cocaine and amphetamine regulated transcript), and *pomc* (proopiomelanocortin) were tested in head segment, The *ghrelin*, *leptin*, *lepr* (leptin receptor), and *cck* (cholecystokinin) were tested in visceral mass. Values are means (*n* = 3), with their standard errors represented by vertical bars. Bars bearing the same or no letters were not significantly different determined by Tukey's test (*P* > 0.05).

For the orexigenic factor genes, with GL level in the micro-diet increasing from 0% to 0.02%, the mRNA expression of *npy* and *agrp* increased followed by decreased (Figure 1). The mRNA expression of *npy* and *agrp* were remarkedly increased in larvae fed the diet with 0.005% GL compared with the control (*P* < 0.05). For the anorexigenic factor genes, with the increase of GL from 0% to 0.02%, the mRNA expression of *trh*, *lepr*, and *cck* showed a decreasing trend. The mRNA expression of *trh* and *lepr* of larvae fed diets with GL was significantly lower than the control, while the mRNA expression of *cck* in larvae fed the diet with 0.02% GL was significantly lower than the control (*P* < 0.05). The mRNA expression of *cart* was significantly decreased in larvae fed diets with GL compared with the control (*P* < 0.05). No significant differences were found among dietary treatments in gene expression of *ghrelin*, *pomc*, and *leptin* (*P* > 0.05).

### 3.3. Digestive Enzymes Activities and Intestinal Development Index

For digestive enzymes activities, with the increase of GL in diets, activities of trypsin, *α*-amylase, and lipase in both PS and IS of larvae increased firstly and then decreased (Table 5). Larvae fed the diet with 0.005% GL had significantly higher activity of trypsin in PS and IS than the control (*P* < 0.05). Meanwhile, larvae fed the diet with 0.005% GL had significantly higher activity of *α*-amylase and lipase in IS than the control (*P* < 0.05). However, no significant differences were found among dietary treatments in activities of *α*-amylase and lipase in larval PS (*P* > 0.05). For intestinal development index, larvae fed diets with GL had significantly higher activity of AKP in brush border membranes than the control (*P* <0.05). No significant differences were detected in the activity of LAP among dietary treatments (*P* >0.05).

### 3.4. Antioxidant Capacity

**Figure 2:**
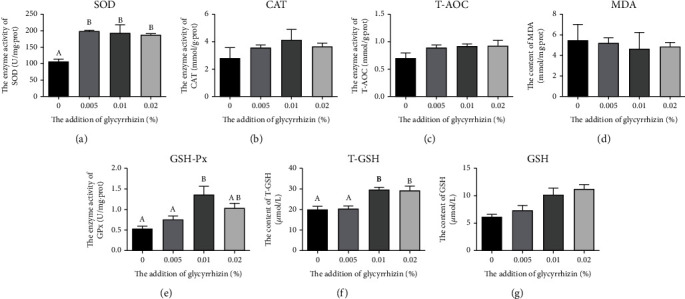
Activities of SOD, CAT, T-AOC, concentration of MDA, activities of GSH-Px, content of T-GSH, and GSH in visceral mass of large yellow croaker larvae. (a) SOD: superoxide dismutase; (b) CAT: catalase; (c) T-AOC: total antioxidant capacity; (d) MDA: malondialdehyde; (e) GSH-Px: glutathione peroxidase; (f) T-GSH: total glutathione; (g) GSH: glutathione. Values are means (*n* = 3), with their standard errors represented by vertical bars. Bars bearing the same or no letters were not significantly different determined by Tukey's test (*P* > 0.05).

With the increase of GL supplementation from 0% to 0.02%, activities of SOD and GSH-Px in larvae showed increasing followed by decreasing trends (Figures 2(a)–2(e)). Compared with the control, larvae fed diets with GL had significantly higher activity of SOD (*P* < 0.05). The activity of GSH-Px in larvae fed the diet with 0.01% GL was remarkedly increased compared with the control (*P* < 0.05). The content of T-GSH in larvae fed diets with 0.01% and 0.02% GL was significantly higher than the control (*P* < 0.05) (Figure 2(f)). However, no statistical discrepancy was found among dietary treatments in T-AOC, CAT, MDA. and GSH (*P* >0.05) (Figures 2(b), 2(c), 2(d), and 2(g)).

### 3.5. Inflammation-Related Indexes

**Figure 3:**
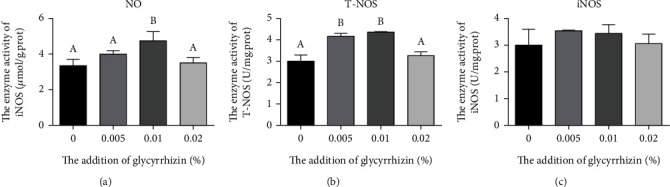
The content of NO and activities of T-NOS and iNOS in visceral mass of large yellow croaker larvae. (a) NO: nitric oxide; (b) T-NOS: total nitric oxide synthase; (c) iNOS: inducible nitric oxide synthase. Values are means (*n* = 3), with their standard errors represented by vertical bars. Bars bearing the same or no letters were not significantly different determined by Tukey's test (*P* > 0.05).

**Figure 4:**
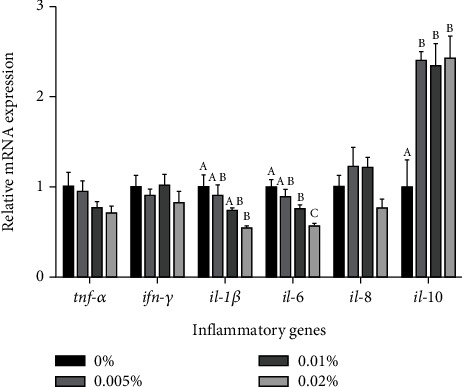
Expression of genes related to inflammation in visceral mass of large yellow croaker larvae. *Tnf-a:* tumor necrosis factor a; *ifn-γ*: interferon *γ*; *il-1β*: interleukin-1*β*; *il-6*: interleukin-6; *il-8*: interleukin-8; *il-10*: interleukin-10. Values are means (*n* = 3), with their standard errors represented by vertical bars. Bars bearing the same or no letters were not significantly different determined by Tukey's test (*P* > 0.05).

With the increase of GL from 0% to 0.02%, the NO content, T-NOS activity, and iNOS activity in larvae ascended firstly and then descended (Figures 3(a)–3(c)). The content of NO in larvae fed the diet with 0.01% GL was remarkedly increased compared with the control. (*P* < 0.05) (Figure 3(a)). Larvae fed diets with 0.005% and 0.01% GL had significantly higher activity of T-NOS than the control (*P* < 0.05). However, there were no significant differences among dietary treatments in iNOS (*P* > 0.05). For the proinflammatory cytokine genes, with increasing GL supplementation, the mRNA expression of *thf-α*, *inf-γ*, *il-1β*, and *il-6* showed decreasing trends, while the mRNA expression of *il-8* increased firstly and then decreased (Figure 4). The mRNA expression of *il-1β* and *il-6* in larvae fed the diet with 0.02% GL were remarkedly decreased compared with the control (*P* < 0.05). However, the mRNA expression of *tnf-a*, *ifn-γ*, and *il-8* showed no significant difference among different treatments (*P* >0.05). For the anti-inflammatory cytokine genes, larvae fed diets with GL had significantly higher mRNA expression of *il-10* than the control (*P* <0.05).

## 4. Discussion

The main components of many Chinese herbal medicines can be used as feed additives to improve the survival and growth performance of cultured animals [8, 10, 32]. The result of this study demonstrated that GL supplementation could increase the survival rate, specific growth rate, and weight gain rate of large yellow croaker larvae. Similarly, the promoting effect of growth performance was also reported in white shrimp [23] and yellow catfish (*Pelteobagrus fulvidraco*) [41]. These results were probably due to the positive effect of GL supplementation on appetite, digestive enzymes activities, antioxidant capacity, and anti-inflammatory capacity.

In the early stage of growth, appetite stimulation plays a vital role in increasing the survival and growth performance of larvae [42, 43]. The appetite of larvae can be regulated by orexigenic factor genes (*npy*, *agrp*, and *ghrelin*) and anorexigenic factor genes (*cart*, *pomc*, *trh, leptin*, *lepr*, and *cck*) [44–46]. The data showed that the supplementation of 0.005% GL could increase the mRNA expression of *npy* and *agrp*, while GL supplementation could decrease the mRNA expression of *trh, cart*, and *lepr*. Similarly, licorice extract supplementation had been confirmed to increase the growth performance in poultry by stimulating digestion and appetite [47]. In general, GL supplementation might stimulate the larval appetite by regulating the mRNA expression of orexigenic and anorexigenic factors, which might increase the growth performance of large yellow croaker larvae.

Digestive enzymes activities are widely used as an index to study the nutritional status of fish larvae. [48–52]. In this study, the supplementation of 0.005% GL could increase the trypsin activity in both pancreatic and intestinal segments, the *α*-amylase activity, and the lipase activity in intestinal segments. Similarly, silver carp (*Hypophthalmichthys molitrix*) fed the diet with GL had higher digestive enzymes activities [24]. Especially, 0.01% GL supplementation could significantly enhance the activities of AKP, which was consistent with previous study of silver carp (*Hypophthalmichthys molitrix*) [24]. In summary, GL supplementation could enhance the digestive capacity of large yellow croaker larvae by increasing digestive enzymes activities.

Larvae are in a period of rapid growth and are prone to oxidative stress, which will produce oxygen free radicals, eventually leading to body damage [53–56]. The antioxidant system of fish can resist oxidative damage and maintain body health [57]. In this study, GL supplementation could increase the SOD activity, which was similar to the results of white shrimp and yellow catfish treated with GL supplementation [23, 41]. This study also demonstrated that the supplementation of 0.01% GL could increase the content of T-GSH and the activity of GSH-Px, which agreed with the previous study on silver carp [24]. In general, GL supplementation could improve antioxidant capacity of large yellow croaker larvae by increasing antioxidant enzymes activities.

The immune system of larvae was undeveloped, which lead to high mortality in the larval stage [58–60]. In the present study, the supplementation of 0.01% GL could increase the NO content and the T-NOS activity of larvae, which was consistent with the survival rate. A similar result also can be found in the study of white shrimp [23]. Cytokines plays a vital role in innate immunity and inflammatory responses [61]. Many studies have proved that GL can stimulate anti-inflammatory factor production and suppress pro-inflammatory factor production from macrophages [61–63]. In this study, the supplementation of 0.02% GL could decrease the mRNA expression of proinflammatory factors (*il-1β* and *il-6*) and increase the mRNA expression of anti-inflammatory factor (*il-10*), which probably due to the higher proportion of GL supplementation could better reflect the anti-inflammatory capacity of GL. Similar results have been confirmed in studies of silver carp [24], Asian seabass [21], blunt snout bream [22], and yellow catfish [41]. In summary, the supplementation of 0.02% GL could alleviate the inflammation of large yellow croaker larvae.

## 5. Conclusion

In conclusion, results of the present study confirmed that the supplementation of 0.005% -0.01% GL could enhance the expression of orexigenic factor genes, activities of digestive enzymes, and antioxidant capacity, while the supplementation of 0.02% GL might alleviate inflammation.

## Figures and Tables

**Table 1:** The formulation and composition of feed trial for large yellow croaker (% dry matter).

Ingredients % dry diet	Microdiets (GL level %)			
	Control (0%)	Diet1 (0.005%)	Diet2 (0.01%)	Diet3 (0.02%)
Whitefish meal^1^	39.77	39.77	39.77	39.77
Krill meal^1^	10.81	10.81	10.81	10.81
Squid meal^1^	4.27	4.27	4.27	4.27
Soy protein concentrate^1^	5.41	5.41	5.41	5.41
Vital wheat gluten^1^	12.84	12.84	12.84	12.84
Yeast hydrolysate^1^	4	4	4	4
Fish oil	8	8	8	8
Ascorbyl polyphosphate	0.2	0.2	0.2	0.2
*α*-Starch	8	8	8	8
Alginae sodium	2	2	2	2
Antioxidant^2^	0.05	0.05	0.05	0.05
Mold inhibitor^3^	0.05	0.05	0.05	0.05
Vitamin premix^1^	1.5	1.5	1.5	1.5
Mineral premix^1^	1	1	1	1
Soybean lecithin^4^	5	5	5	5
GL^5^	0	0.005	0.01	0.02
Microcrystalline cellulose^6^	0.04	0.035	0.03	0.02
Choline chloride	0.2	0.2	0.2	0.2
Composition % dry diet				
Crude protein	54.38	53.99	54.06	53.78
Crude lipid	16.46	16.63	16.19	16.31
Ash	12.12	12.34	12.62	12.27

^1^ Ingredients purchased from Great Seven Biotechnology Co., Ltd in Shandong, China. The composition of elementary ingredients, vitamin premix, and mineral premix refers to Huang et al. [38]. ^2^ The antioxidant is ethoxy quinoline. ^3^ The mold inhibitor is calcium propionate. ^4^ Commercially available from Beijing Huaxia Houde Co., Ltd. (Beijing, China). ^5^ The pure glycyrrhizin was purchased from Qingdao kaitaike industry and Trade Co., Ltd in Qingdao China. ^6^ Low level of microcrystalline cellulose had no significant effect on large yellow croaker larvae.

**Table 2:** Primer sequences of RT-qPCR used in this study.

Gene	Forward (5′-3′)	Reverse (5′-3′)	Reference
*Npy*	AAAGAGGTCCAGTCCTGAGATT	GTGGCGGCTCATAGTGGTAA	XM019275410
*Agrp*	GAGAGACAAGTGAGAGTTACCG	CGAAGAAGAAAGACGCAG	XM027281261.1
*Trh*	CGACGATGGAGACTGGGATA	CCTCGGCATGACTCTTGTTA	XM010755325.3
*Ghrelin*	TGACCTTGTGGTGCAAGTCGAC	CCGATTTCAAAGGGGGCACT	KC899122
*Cart*	GCCAACTGCAACTCCTACCT	TGCTACAGTAAATAAGCGGAAC	XM010741284.3
*Leptin*	GATGTTCTGGATGGACCTGC	AGACACCACTGATGCGGACT	XM010729765
*Pomc*	AGCCTGATTATCTGCCTCCC	CTTGTACGTGCCGTCCTTCT	XM027291739.1
*Lepr*	CAGCCCATTCATCTCCATTA	CTTGCCTCCTCTTCGTCTTC	XM019270027.2
Cck	TGGCTCCTCACTGTCTCACA	TTGCCTCAACAGACCCTGAT	XM019255482.2
*Ifn-γ*	TCAGACCTCCGCACCATCA	GCAACCATTGTAACGCCACTTA	XM019258900
*Tnf-a*	ACACCTCTCAGCCACAGGAT	CCGTGTCCCACTCCATAGTT	NM001303385
*Il-1β*	CATAGGGATGGGGACAACGA	AGGGGACGGACACAAGGGTA	XM010736551
*Il-6*	CGACACACCCACTATTTACAAC	TCCCATTTTCTGAACTGCCTCT	XM010734753
*Il-8*	AATCTTCGTCGCCTCCATTGT	GAGGGATGATCTCCACCTTCG	XM010737667.3
*Il-10*	AGTCGGTTACTTTCTGTGGTG	TGTATGACGCAATATGGTCTG	XM010738826
*β-Actin*	GACCTGACAGACTACCTCATG	AGTTGAAGGTGGTCTCGTGGA	GU584189

*npy*: neuropeptide Y; *agrp*: agouti-related protein; *trh*: thyrotropin-releasing hormone; *cart*: cocaine and amphetamine-regulated transcript; *pomc*: proopiomelanocortin; *lepr*: leptin receptor; *cck*: cholecystokinin; *ifn-γ*: interferon *γ*, *tnf-a*: tumor necrosis factor *a*; *il-1β*: interleukin-1*β*; *il-6*: interleukin-6; *il-8*: interleukin-8; *il-10*: interleukin-10.

**Table 3:** Effects of GL supplementation on survival and growth performance of large yellow croaker larvae.

Parameters	Microdiets (GL level%)			
	Control (0%)	Diet1 (0.005%)	Diet2 (0.01%)	Diet3 (0.02%)
Final body length (mm)	18.33 ± 2.70^a^	22.60 ± 2.72^b^	22.26 ± 2.76^b^	21.24 ± 2.51^b^
Final body weight (mg)	137.44 ± 28.48^a^	236.25 ± 27.23^c^	186.22 ± 27.49^b^	170.11 ± 43.44^b^
Specific growth rate (% day^−1^)	11.98 ± 0.18^a^	13.39 ± 0.05^c^	12.99 ± 0.11^bc^	12.68 ± 0.29^b^
Survival rate (%)	8.20 ± 0.73^a^	14.55 ± 2.00^b^	16.44 ± 1.78^b^	14.33 ± 2.12^b^
Weight gain rate (%)	35.40 ± 1.95^a^	54.60 ± 0.85^c^	48.31 ± 1.65^b^	44.04 ± 3.87^b^

Data were expressed as means ± S.E.M, *n* = 3. Means in the same column sharing a same superscript letter are not significantly different determined by Tukey's test (*P* > 0.05).

**Table 4:** Effects of GL supplementation on body composition of large yellow croaker larvae.

Parameters	Microdiets (GL level%)			
	Control (0%)	Diet1 (0.005%)	Diet2 (0.01%)	Diet3 (0.02%)
Moisture	86.87 ± 0.97	$\underset{¯}{86.35\pm 0.40}$	86.75 ± 1.35	86.31 ± 0.49
Protein	7.10 ± 0.20	7.85 ± 0.27	7.72 ± 0.36	8.23 ± 0.42
Lipid	3.02 ± 0.26	3.07 ± 0.34	3.05 ± 0.16	2.99 ± 0.16

Data were expressed as means ± S.E.M, *n* = 3. Means in the same column sharing no superscript letter are not significantly different determined by Tukey's test (*P* > 0.05).

**Table 5:** Effects of GL supplementation on activities of digestive enzymes of large yellow croaker larvae.

Parameters		Experimental diets (GL level)			
		Control (0%)	Diet1 (0.005%)	Diet2 (0.01%)	Diet3 (0.02%)
Trypsin	PS^2^	66.91 ± 8.01^a^	85.76 ± 2.38^b^	64.86 ± 13.32^a^	61.02 ± 8.78^a^
	IS^2^	70.65 ± 16.74^a^	119.85 ± 22.44^b^	82.38 ± 21.82^a^	81.22 ± 17.57^a^

*α*-Amylase	PS	0.20 ± 0.05	0.28 ± 0.01	0.19 ± 0.03	0.28 ± 0.04
	IS	0.07 ± 0.03^a^	0.25 ± 0.11^b^	0.10 ± 0.06^ab^	0.08 ± 0.02^a^

Lipase	PS	66.56 ± 15.16	78.35 ± 29.25	60.30 ± 16.99	68.62 ± 16.99
	IS	64.88 ± 13.62^a^	143.14 ± 20.95^b^	66.02 ± 18.87^a^	86.85 ± 16.71^a^

LAP	BBM^2^	1.43 ± 0.34	1.84 ± 0.58	2.09 ± 0.61	1.52 ± 0.40

AKP	BBM	25.60 ± 12.56^a^	37.18 ± 6.85^a^	77.90 ± 10.21^b^	24.52 ± 8.32^a^

Data were expressed as means ± S.E.M, *n* = 3. Means in the same column sharing a same or no superscript letter are not significantly different determined by Tukey's test (*P* > 0.05). ^2^ PS: Pancreatic segments; IS: Intestinal segments; BBM: Brush border membranes.

## Data Availability

The data that support the findings of this study are available from the corresponding author upon reasonable request.
